# Efficacy and Safety of Auranofin for Progressive Desmoid-Type Fibromatosis: The Study Protocol of an Open-Label Phase II Trial

**DOI:** 10.7759/cureus.71033

**Published:** 2024-10-07

**Authors:** Yoshihiro Nishida, Kan Ito, Tomohisa Sakai, Fumie Kinoshita, Yachiyo Kuwatsuka, Saori Kinoshita, Shiro Imagama

**Affiliations:** 1 Rehabilitation Medicine, Nagoya University Hospital, Nagoya, JPN; 2 Orthopedic Surgery, Nagoya University Hospital, Nagoya, JPN; 3 Orthopedic Surgery, Nagoya University Graduate School of Medicine, Nagoya, JPN; 4 Advanced Medicine, Nagoya University Hospital, Nagoya, JPN; 5 Hospital Pharmacy, Nagoya University Hospital, Nagoya, JPN

**Keywords:** auranofin, clinical trial, desmoid, drug repositioning, protocol

## Abstract

Background

As desmoid-type fibromatosis (DF) exhibits a high recurrence rate after surgery, initial active surveillance followed by medical therapy is the mainstay of the treatment. However, there are few effective drugs with acceptable side effects.

Methodology

Among drugs that have been used for a long period and possess a known safety profile, auranofin was observed to be effective in suppressing DF using the drug repositioning method in our laboratory. This clinical study has been designed to examine the efficacy and safety of auranofin, an approved anti-rheumatic drug, in patients with progressive DF.

Results

This study is conducted as a single-center, single-arm, open-label study. Auranofin 3 mg tablets will be administered twice daily to DF patients with progressive disease. The primary endpoint is progression-free survival at 26 weeks after starting treatment. Secondary endpoints include response rate, T2-weighted MRI evaluation, pain intensity, quality of life (QOL), and safety assessment.

Conclusions

This is the first clinical trial of auranofin in patients with aggressive DF. The study will allow an in-depth understanding of the efficacy of auranofin for response rate as well as for changes in MRI findings, pain, and QOL in patients with aggressive DF.

## Introduction

Desmoid-type fibromatosis (DF) is a (myo-)fibroblast-proliferating soft tissue tumor that is classified as an intermediate locally aggressive tumor in the new 2020 WHO classification [1]. DF is a rare disease that occurs in 2.4-4.3 people per million individuals per year, and the site of occurrence is generally divided into intraperitoneal and extraperitoneal tumors. The majority of extraperitoneal tumors are thought to be caused by mutations in the *β-catenin* gene and commonly affect the abdominal wall, shoulder girdle, buttocks, and extremities [2]. DF that occurs in familial adenomatous polyposis patients often arises in mesenteric or retroperitoneal regions and possesses an adenomatous polyposis coli germline mutation [3].

Among bone and soft tissue tumors classified as intermediate types, DF is characterized by a significantly high postoperative recurrence rate and spontaneous regression in some cases. Additionally, in numerous cases, activities of daily living (ADLs) and quality of life (QOL) may worsen due to joint contracture and pain caused by DF during follow-up and after surgery [4]. Based on these findings, the standard treatment for DF is surgical treatment aiming for an R0 resection; however, the mainstay of treatment becomes active surveillance involving careful follow-up without any active therapeutic intervention. Experts from Europe, America, and Asia have published a statement of treatment policy for DF based on past literature reviews [5]. In this manuscript, the first option in the treatment algorithm for DF is active surveillance. However, there are a significant number of cases in which DF cannot be controlled with active surveillance. In such cases, implementation of active treatment is necessary to control the disease [6,7]. Specifically, there are cases in which a drug with evidence of effectiveness is required.

Drugs that have long been used to treat DF include non-steroidal anti-inflammatory drugs (NSAIDs), antihormonal drugs, anticancer drugs, and molecular target drugs [8]. Among these, certain anticancer drugs and molecular target drugs have been reported to be effective [5]. Combination therapy involving the use of methotrexate (MTX) and vinblastine (VBL) has long been used as an anticancer therapy. Azzarelli et al. administered MTX 30 mg/m^2^ + VBL 6 mg/m^2^ once each week to 30 DF patients and observed that partial response was achieved in 12 (40%) patients and stable disease in 18 patients. Conversely, serious bone marrow suppression was reportedly observed in four (13%) patients as an adverse event [9]. The results of combined therapy of MTX and VBL have been reported for 38 cases of DF in Japan, and the clinical benefit rate was 95%. Progression-free survival (PFS) at five years was 80.8%. However, three cases of grade 3/4 adverse events were observed [10]. The effectiveness of doxorubicin-based chemotherapy has also been reported. However, considering that DF is not a malignant tumor, many facilities use doxorubicin treatment which is known to elicit strong side effects as a third-line drug treatment [8].

Several molecular-targeted drugs have also been demonstrated to be effective against DF. Gounder et al. conducted a double-blind randomized controlled trial reporting the efficacy of sorafenib for progressive DF [11]. The median follow-up period in this trial was 27 months, and the two-year PFS rate was 81% in the sorafenib group and 36% in the placebo group. These rates were statistically significant, thus demonstrating the efficacy of sorafenib (p < 0.001). In the sorafenib group, the response rate was 33%, and the median time to tumor shrinkage and evaluation was 9.6 months. Toulmonde et al. reported the results of a multicenter, phase II, randomized, open-label study of pazopanib versus MTX + VBL for progressive DF (DESMOPAZ study) [12]. In this study, a one-year PFS rate of 85.6% was reported in the pazopanib group. The main side effects of pazopanib were hypertension and diarrhea. Recently, the effectiveness of nirogacestat in progressive DF has been reported [13]. However, these molecular-targeted drugs are expensive and cause significant side effects.

Considering the current state of drug treatment for DF, a study was conducted using drug repositioning techniques to discover new drugs that elicit few side effects and are relatively inexpensive. Using Prestwick’s Chemical Library which contains 1,186 off-patent pharmaceutical products that comprehensively cover a wide range of disease areas, we screened drugs that are effective against DF in an in vitro experimental system using several DF cell lines. The antirheumatic drug auranofin was identified as exerting anti-DF tumor effects. The antitumor effect of this drug was also observed in vivo using a mouse model of spontaneous DF onset [14].

Auranofin has long been used to treat rheumatoid arthritis and possesses a proven safety profile for long-term use. As it is inexpensive and causes few side effects, we decided to plan the first clinical trial of auranofin in patients with progressive DF.

## Materials and methods

Study design

The study design will be a single-center, single-arm, open-label exploratory study. This clinical study will be conducted at one facility, the Nagoya University Hospital.

Study objective

**Figure 1:**
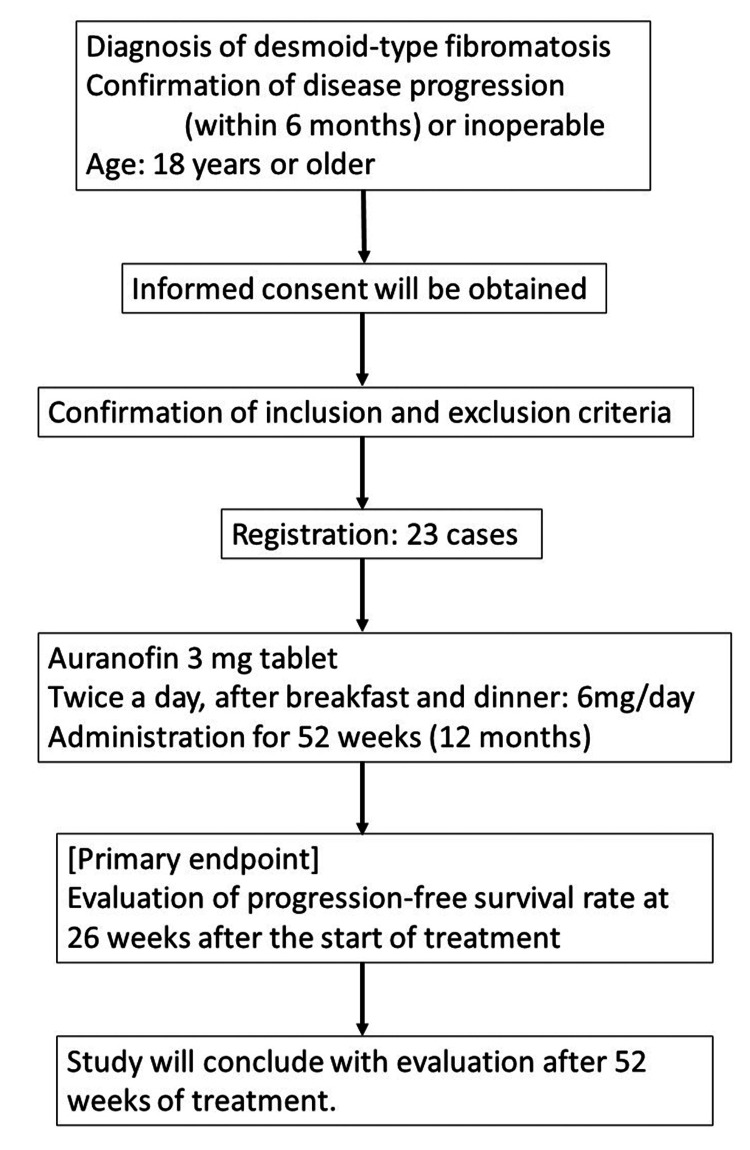
Flowchart of the study procedure.

This study will be the first clinical trial to examine the efficacy and safety of auranofin, an antirheumatic drug already approved, in patients with DF (Figure 1).

Endpoints

The primary endpoint will be PFS at 26 weeks after the start of treatment. The secondary endpoints will be the following: (i) to (vi) at 12 and 52 weeks after the start of treatment; (i) to (v) at 26 weeks; the best response during the study period (Table 1). Throughout the study period, safety will be assessed according to the Common Terminology Criteria for Adverse Events, version 5.0.

**Table 1:** Study endpoints.

Endpoints
(i) Response rate (MRI or CT according to the Response Evaluation Criteria in Solid Tumors (RECIST ver. 1.1): CR (complete response)/PR (partial response)/SD (stable disease) rate
(ii) Percentage of high-intensity areas in T2-weighted MRI
(iii) Pain scale assessment using the Numerical Rating Scale (NRS)
(iv) Quality of life (QOL) assessment using the EuroQol 5-dimensional questionnaire (EQ-5D)
(v) GODDESS (Gounder/DTRF Desmoid symptom/Impact Scale [15], NIH-Developed QOL Assessment for DF)
(vi) Progression-free survival

Eligibility criteria

Inclusion and Exclusion Criteria

Patients who meet all of the conditions listed in Table 2 will be selected, and those who meet one of the conditions listed in Table 3 will be excluded.

**Table 2:** Inclusion criteria.

Inclusion criteria
(i) Patients who have been definitively diagnosed with desmoid-type fibromatosis (DF) by the time of consent. Both the sporadic type and the familial adenomatous polyposis type are acceptable
(ii) Patients aged 18 years or older at the time of consent
(iii) Patients with evaluable lesions
(iv) Patients with tumor progression within six months prior to the start of the clinical study or patients with inoperable disease and worsening functional disability
(v) Patients who have provided written informed consent
(vi) Patients with Eastern Cooperative Oncology Group (ECOG) Performance Score of 0 or 1 within 14 days prior to enrolment
(vii) Test results within 14 days prior to registration indicate adequate function of vital organs, including bone marrow, liver, and kidney
(viii) Appropriate cardiac function and normal waveforms on a 12-lead electrocardiogram

**Table 3:** Exclusion criteria.

Exclusion criteria
(i) Patients for whom auranofin is contraindicated
(ii) Patients who have taken auranofin within 4 weeks prior to obtaining consent
(iii) Patients who have taken anticancer drugs or molecular-targeted drugs within 4 weeks prior to obtaining informed consent
(iv) Patients who are deemed ineligible for enrolment in the clinical study based on the principal investigator (sub-investigator) believing that participation in the clinical study or administration of the test drug will increase their risk
(v) Patients for whom MRI and CT are not suitable
(vi) Patients with difficulty taking oral medications
(vii) Patients otherwise deemed inappropriate for the clinical study by the principal investigator or co-investigator

Study discontinuation criteria

This study will be discontinued for patients who meet one of the conditions listed in Table 4.

**Table 4:** Criteria for study discontinuation.

Study discontinuation criteria
(i) If the patient wishes to discontinue
(ii) If a safety issue such as a serious illness occurs
(iii) If adverse events of Common Terminology Criteria for Adverse Events (CTCAE) Grade 3 or higher occur, treatment will be suspended. If the event improves within 6 weeks, treatment will be resumed. If the event does not improve within 6 weeks, treatment will not be resumed
(iv) If efficacy issues such as no improvement in the target disease arise
(v) If a serious event occurs that requires surgical intervention
(vi) If the patient becomes pregnant
(vii) If the patient does not come to the hospital due to relocation or other reasons
(viii) Any other circumstances that require the suspension or interruption of a part or the whole of a clinical study occur

## Results

Registration

Once inclusion and exclusion criteria would be confirmed and written informed consent would been obtained, patients will be registered through the RedCap system by their treating physician.

Treatment procedure and evaluation

Auranofin 3 mg tablets will be administered orally twice daily after breakfast and dinner for 52 weeks until disease progression, death, or intolerable side effects are observed or until the patient wishes to discontinue the clinical study for other reasons. This dose is equivalent to that used in preclinical in vivo studies. The outcome measures, clinical tests, and evaluation schedule are presented in Table 5.

**Table 5:** Schedule of the outcome measures, clinical tests, and evaluation. IC: informed consent; QOL: quality of life; GODDESS: GounderDTRF Desmoid symptom/Impact Scale; PS: performance status; ECG: electrocardiogram

	Pre-registration	Treatment period								Completion	
		Day 1～Week 12						≥Week 12 (2)			
	≤14 days	Day 1 (1)	Week 4	Week 8 (Day 57)	Week 12 (Day 85)	Week 18 (Day 127)	Week 26 (Day 183)	Week 34 (Day 239)	Week 42 (Day 295)	Week 52 (Day 365)	Cessation
			(Day 29)								
Allowable days	‒	‒	±8 days	±8 days	±8 days	±8 days	±14 days	±8 days	±8 days	±8 days	±28 days
Written IC	●										
Screening											
Case selection	●										
Disease evaluation											
Image	●				●		●			●	●
Pain	●		●	●	●	●	●			●	●
(NRS)											
QOL (EQ-5D, GODDESS)	●		●	●	●	●	●	●	●	●	●
Safety assessment											
PS	●	●	●	●	●	●	●	●	●	●	●
Vital signs	●	●	●	●	●	●	●	●	●	●	●
Adverse events			●	●	●	●	●	●	●	●	●
Clinical laboratory tests											
Biochemical testing	●		●	●	●	●	●	●	●	●	●
Hematological tests	●		●	●	●	●	●	●	●	●	●
Urine tests	●		●	●	●	●	●	●	●	●	●
ECG	●						●			●	●

Day 1 is the start of treatment. Before administration, the patient’s overall condition and vital signs should be evaluated. Even after the primary endpoint is confirmed at Week 26, treatment will generally continue until Week 52, as this is the treatment period. Disease evaluation will continue until the diagnosis of definite disease progression or for the duration of the 52-week clinical study. Radiological evaluation of pre-enrollment examinations will be allowed within four weeks of starting the study drug administration. T1 and T2-weighted MRI images, including target lesions, will be obtained. If an MRI cannot be taken, contrast CT will be taken. The patient’s survival/death and outcome investigation date will be recorded in the case report form. Biochemical tests will be performed on the specified date to assess total protein, albumin, total bilirubin, aspartate aminotransferase, alanine transaminase, alkaline phosphatase, serum creatinine, blood glucose, electrolytes, lipase, and amylase. Hematological tests will be performed on the specified days to assess red blood cell count, white blood cell count, differential white blood cell count, platelet count, hemoglobin, and hematocrit. Urine tests will be performed on the specified days to assess protein, occult blood, and sediment. During continued administration, all illnesses and serious illnesses will be monitored until recovery, stabilization, loss of follow-up, or death is confirmed.

## Discussion

Follow-up

After the trial is completed at 52 weeks or discontinued at the patient’s request, routine medical care for DF will be provided.

Sample size estimation and statistics

In a previous report, in a phase III, double-blind, randomized controlled trial of sorafenib, the one-year PFS rate was 89% in the sorafenib group and 46% in the placebo group [11] Additionally, in a phase II, randomized, non-comparative trial of pazopanib and MTX + VBL combination therapy, the primary endpoint of the six-month PFS rate was 83.7% in the pazopanib group and 45.0% in the MTX + VBL group [12]. If an effect similar to that of these drugs is observed, it is considered clinically significant. The number of cases required for auranofin administration was calculated to be 21 while assuming a threshold PFS rate of 40% at six months, an expected PFS rate of 70%, a significance level of 10% on both sides, and a detection power of 80%. Assuming that 10% (two cases) would drop out, the target number of cases is 23.

Statistical methods used will include descriptive statistics, Student’s t-test, and Fisher’s exact test as appropriate. The Kaplan-Meier method will be used to analyze survival curves.

Institutional review board approval and registration of the study

The study protocol was approved by the Nagoya University Certified Review Board (number 2021-0259). The study is registered in the Japan Registry of Clinical Trials (jRCT) as jRCTs041210071 (https://jrct.niph.go.jp/). More details about this study can be found on the jRCT website (https://jrct.niph.go.jp/latest-detail/jRCTs041210071). The study will be conducted in accordance with the Declaration of Helsinki, the Clinical Trials Act, and other current legal regulations in Japan.

## Conclusions

This is the first clinical trial of auranofin in patients with progressive DF detected by drug repositioning. This study will provide a detailed understanding of the efficacy of auranofin on response rates, MRI findings, pain, and changes in QOL in patients with progressive DF. The results will contribute to the development of less toxic and less costly drugs.
